# Butyrophilins: γδ T Cell Receptor Ligands, Immunomodulators and More

**DOI:** 10.3389/fimmu.2022.876493

**Published:** 2022-03-17

**Authors:** Thomas Herrmann, Mohindar M. Karunakaran

**Affiliations:** Institute for Virology and Immunobiology, Julius Maximilians Universität Würzburg, Würzburg, Germany

**Keywords:** butyrophilin, immune therapy, T cell receptor, γδ T cell, BTN3A1, BTN2A1, phosphoantigen, tumor

## Abstract

Butyrophilins (BTN) are relatives of the B7 family (e.g., CD80, PD-L1). They fulfill a wide range of functions including immunomodulation and bind to various receptors such as the γδ T cell receptor (γδTCR) and small molecules. One intensively studied molecule is BTN3A1, which binds *via* its cytoplasmic B30.2 domain, metabolites of isoprenoid synthesis, designated as phosphoantigen (PAg), The enrichment of PAgs in tumors or infected cells is sensed by Vγ9Vδ2 T cells, leading to the proliferation and execution of effector functions to remove these cells. This article discusses the contribution of BTNs, the related BTNL molecules and SKINT1 to the development, activation, and homeostasis of γδ T cells and their immunomodulatory potential, which makes them interesting targets for therapeutic intervention.

## What Are Butyrophilins?

**Figure 1:**
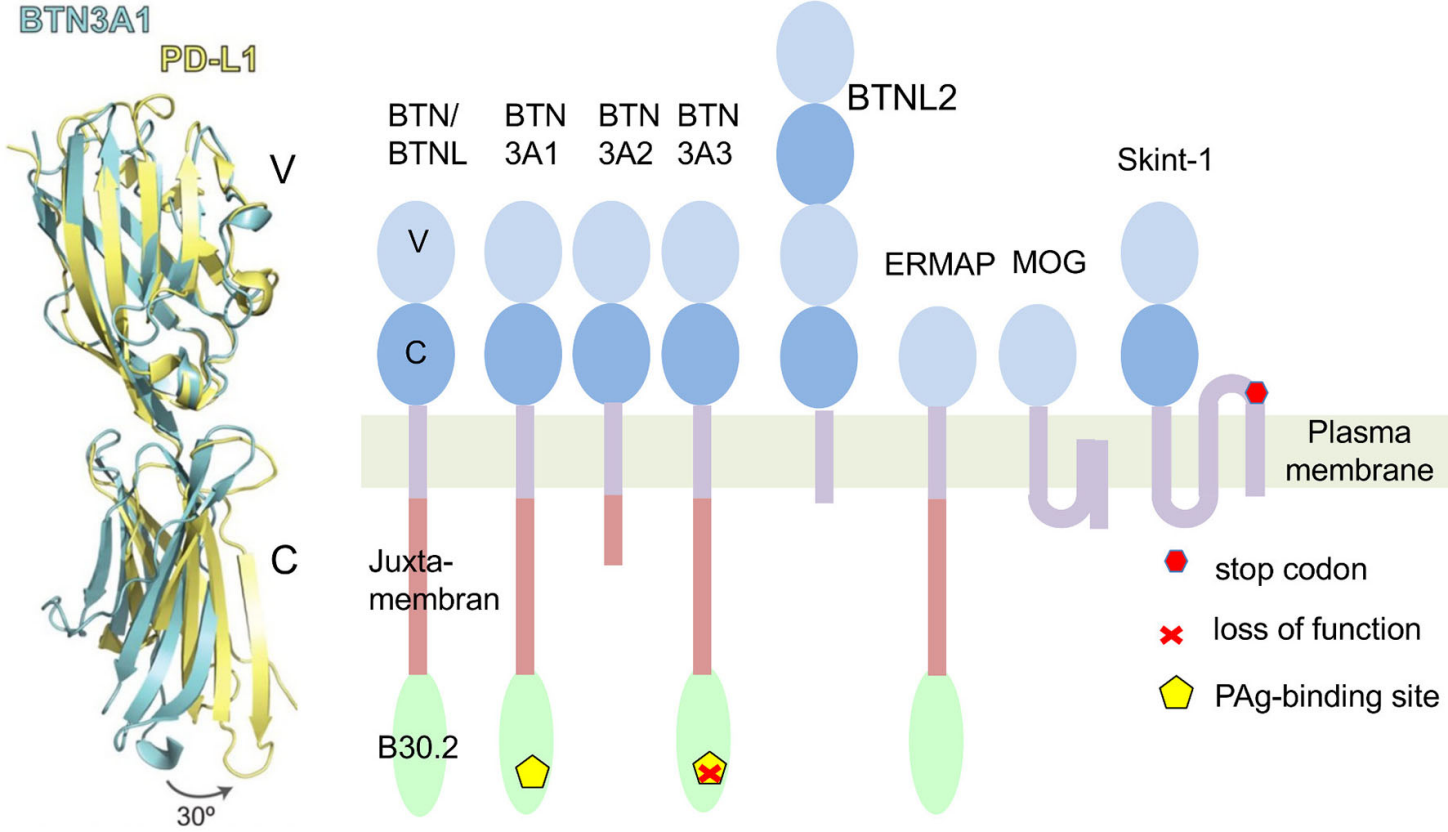
Butyrophilins as members of the B7 family. Left: A comparison of structures of extracellular domains of programmed death ligand 1 (PD-L1: yellow) and Butyrophilin 3A1 (BTN3A1: blue). Shown is an overlay of the tertiary structure of both proteins. Differences of the C-domains of both molecules result mainly from a twist in the orientation of V- and C-Ig domains. In an exclusive overlay of V domain alone, similarities in the Ig-V domains would be even more pronounced. Picture is modified from (3). Right: Schematic representation of human BTN/BTNL family members and mouse Skint1. The protein domains are exemplified for BTN3A1. ERMAP (Erythroblast membrane-associated protein), MOG (Myelin-Oligodendrocyte glycoprotein). Skint 1 (Selection and upkeep of intraepithelial T-cells 1). The indicated Stop codon inactivates the Skint1 function in the DETC deficient FVB/N-Tac mouse strain (4).

Eponymous for the butyrophilin family is butyrophilin 1A1 (BTN1A1 in humans, Btn1a1 in mice). It controls the fat content of milk and is found in membranes of milk-secreting mammary gland epithelial cells and fat droplets (1). Interestingly, it is also expressed in the thymic epithelium (2). The extracellular domains of BTN1A1 and other members of the BTN-family share structural features with the B7 family (**Figure 1**) (5). Its cytoplasmic region contains a juxtamembrane coiled-coiled domain and a B30.2 domain. B30.2 domains are also found in many members of the “tripartite-motif” family (TRIM), known to be involved in innate immune responses and serve as platforms for interaction with other proteins (6). The human *BTN* genes are all part of the gene cluster located at the telomeric end of the *MHC* complex on chromosome 6. It contains protein-encoding genes (7). Four of them; BTN2A1, BTN3A1 (CD277), BTN3A2 and BTN3A3, contribute to γδ T cell activation (8–13). While BTN3 genes first emerged in placental mammals (14) BTN1 and BTN2 genes co-emerged with the evolvement of vertebrates (7).

## Butyrophilins and γδ T Cell Development

Some BTN-, Butyrophilin like (BTNL) and the BTN-related Skint (Selection and upkeep of intraepithelial T cells) molecules control the development and function of γδ T cells (5). γδ T cells are “non-conventional” T-lymphocytes, which are defined by expression of the γδ T cell antigen receptor (γδTCR). They can be found in nearly all jawed vertebrates. γδ T cells differ profoundly from MHC restricted αβ T cells in antigen-recognition, thymic development and selecting ligand. In contrast to MHC restricted αβ T cells, whose TCR bind with the CDRs (complementarity determining region) 1, 2 and 3 of both the TCRα and β chains to MHC-peptide complexes, γδTCRs do not share specificity for a common class of ligands. The known γδTCR antigens are soluble molecules such Phycoerythrin, various bacterial proteins and stress-induced MHC- and MHC-like molecules (15). γδ T cells differ from MHC restricted T cells in thymic development, notably that for some populations of γδ T cells the strength of TCR-ligand interaction during thymic development does not result in classical positive or negative selection, but in programming for differentiation to IFNγ or IL-17-producing cells (16, 17). Furthermore, γδTCR rearrangements are typical for certain phases of thymic development and lead to highly specialized γδ T-cell populations. The most prominent example for such a development is the exclusive homing and functional specialization of the DETC (Dendritic Epidermal T cells) which help to maintain the barrier function of the skin (18). They are the first T cell population to develop in a body and are characterized by their dendritic morphology and a uniform TCR. The discovery of a mouse strain variant devoid of these cells led to the discovery of the butyrophilin-related gene *Skint1* gene (4) which together with Skint2 is essential for DETC development (19). Classical DETC has been found so far only in rodents, but interestingly DETC-resembling cells with variable γδ TCR exist in the crab-eating macaques (*Macaca fascicularis*) which, in contrast to the DETC negative hominids, carry a functional *SKINT1L* (Skint1 like) gene (18).

In humans, most of the circulating γδ T cells contain a Vδ2-bearing δ chain, while TCR of resident human γδ T cells are dominated by other Vδ (20). Some BTNs and BTNL molecules control γδ T cell subset homeostasis and activation and bind to their TCR (21).

BTN and BTNL proteins are structurally similar, but the composition and chromosomal location of the gene families varies between species. In humans and mice, activation of γδ T cells and homeostasis of populations of intestinal epithelial γδ T cells are under the control of BTN and BTNL molecules. Murine Btnl1-Btnl6 heterodimers interact with Vγ7 positive γδ T cells, and human BTNL3-BTNL8 heterodimers bind the TCR of Vγ4 positive cells. In the latter case, direct binding to germline-encoded parts of the γ-chain, especially to its hypervariable region 4 (HV4/CDR4), has been demonstrated, showing similarities to the interaction of some superantigens with Vβ-gene-encoded regions of αβTCRs (22–24). It is likely that differential topologies of TCR-ligands prompt unique modes of signaling, and it has been hypothesized that this superantigen-like type of binding maintains local cell homeostasis, while binding to the CDR3s supports an antigen-specific immune response (21). Insights to the physiological significance of BTNL-TCRγ interaction can be expected from a rather frequent copy number variation, which generates a BTNL3*8 fusion product not expected to bind to Vγ4TCRs (25).

## Phosphoantigen-Reactive Vγ9Vδ2 T Cells and Butyrophilins

BTN2A1 and BTN3A1 are mandatory for the activation of T cells carrying the eponymous Vγ9Vδ2TCR (10) with a Vγ9JP rearrangement and Vδ2-containing δ-chains. 1-5% of all blood T cells are Vγ9Vδ2 T cells. Usually, Vγ9Vδ2 T cells are cytotoxic cells with a type I cytokine profile, but they also show a remarkable functional plasticity and are activated by pyrophosphorylated metabolites of isoprenoid synthesis, the so-called phosphoantigens (PAgs). One such PAg is isopentenyl diphosphate (IPP), which is found in all organisms. Another well-studied PAg named (E)-4 Hydroxy-3-Methy-but-2-enyl diphosphate (HMBPP) stimulates 10000-fold better than IPP. It is the precursor of IPP in the DOXP metabolic pathway for isoprenoid synthesis, common to many Eubacteria (e.g., Mycobacteria), Apicomplexa such *Plasmodium* spp. or *Toxoplasma gondii* and chloroplasts. HMBPP initiates the massive expansion of the Vγ9Vδ2 T cell population during infections with HMBPP-producers, which can end up with up to 50% of human blood T cells becoming Vγ9Vδ2 T cells. In some tumor cells IPP levels are increased and trigger their elimination *via* Vγ9Vδ2 T cells. Increased IPP levels and concomitant Vγ9Vδ2 T cell activation are also induced by amino-bisphosphonates such as zoledronate, drugs commonly used in the treatment of bone metastasis or osteoporosis (26).

A breakthrough for the understanding of PAg-induced Vγ9Vδ2 T cell activation, was the finding that in cultures with peripheral blood mononuclear cells, monoclonal antibodies against BTN3A stimulated Vγ9Vδ2 T cells (agonist) or inhibited their PAg-response (antagonists) (**Figure 1**). This effect was dependent on the monoclonal antibodies binding to BTN3A molecules expressed by antigen-presenting or tumor cells, and not to the BTN3A molecules on the Vγ9Vδ2 T cells (8). PAg does not bind to the Vγ9Vδ2TCR but needs “presentation” by other cells that is initiated by binding to the intracellular B30.2 domain of BTN3A1 (9). The PAg-binding induces a conformational change which leads to the formation of a BTN3A1-BTN2A1 complex and to a not yet understood change at the cell surface, which is recognized by the Vγ9Vδ2 T cells (27). Whether recognition involves direct binding of BTN3A1 to the TCR or a hypothetical counter receptor on the T cells, or both, is unclear, but the key role of both proteins in PAg-stimulation is undisputed. The function of the PAg-non-binding BTN3A2 and BTN3A3 molecules is to increase the efficacy of the BTN3A1 action in PAg-mediated stimulation (23).

Insights into the mechanism of PAg mediated Vγ9Vδ2 T cell activation came from the comparison of species. The alpaca, beside humans and primates, is one of the few mammals with functional *BTN3*, *TCRVγ9* (*TRGV9*) and *TCRVδ2* (*TRDV2*) genes (14, 28, 29), and possesses PAg reactive Vγ9Vδ2 T cells (14). Replacing the intracellular region of human BTN3A1 with that of an alpaca BTN3 results in a chimeric BTN3 molecule which stimulates as efficiently as the complex of the different human BTN3A molecules (14). Sequence comparison and phylogenetic considerations led to the postulation of an Alpaca-like primordial BTN3 which amalgamates the function of the human BTN3A family and can be imagined as a BTN3A3-like molecule with an intact PAg-binding site (15, 29).

Another example of how a comparison of species’ differences helps for a better understanding of PAg stimulation, is the comparison of rodent cell lines expressing human BTN3A1 with cell lines carrying single human chromosomes. The comparison showed that, together with BTN3A1, other genes on human chromosome 6 are mandatory for PAg presentation (30). Our strategy to identify the gene(s) was the generation of “radiation hybrids” by fusing irradiated human chromosome 6-bearing rodent cells with other rodent cells (11). The chromosomes of the non-irradiated fusion partner randomly integrate pieces of the chromosomes of the irradiated cells including those of chromosome 6. The hybrids were tested, and it was assumed that only cells carrying the missing (human) genes can present PAgs. Comparison of human genome fragments of the PAg-presenting hybrids led to the identification of a 150 kB fragment at the telomeric end of the HLA complex, which contained the entire *BTN* cluster including *BTN2A1*. The significance of BTN2A1 for PAg presentation was demonstrated by:

1. Co-expression of BTN2A1 and BTN3A1 in rodent cells which rendered the cells PAg-presenters.
2. BTN2A1 inactivation by CRISPR-Cas9-mutagenesis of human 293T cells abolished their PAg-presentation capacity.

**Figure 2:**
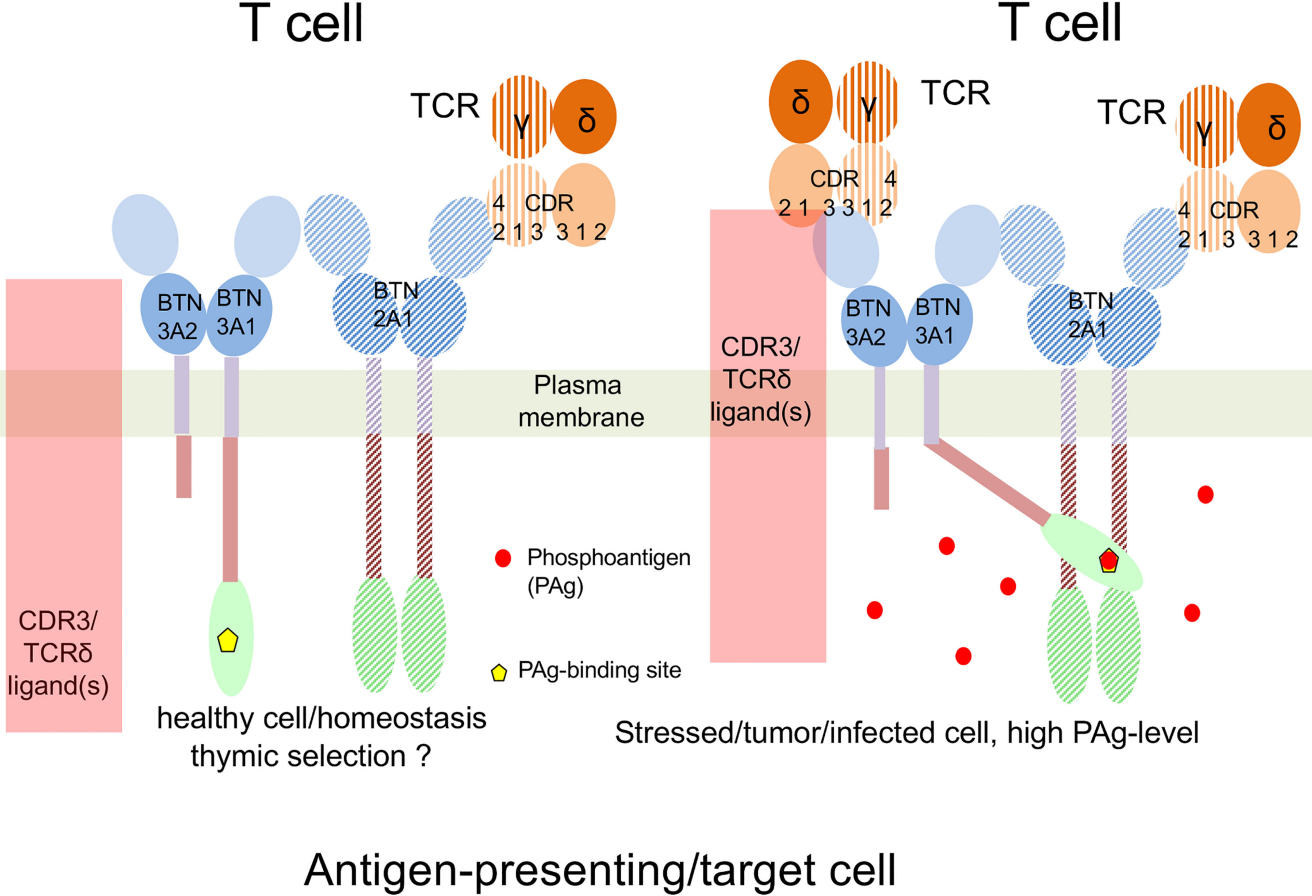
Working hypothesis on PAg-recognition by Vγ9Vδ2 T cells. Left: Under physiological conditions, BTN2A1 binds to the hypervariable region 4 (CDR4 or HV4) of the Vγ9 region of the Vγ9Vδ2TCR. This controls thymic development and homeostasis of Vγ9Vδ2 T cells. Right: Infection or cell transformation increases PAg-levels in the presenting or tumor cell. This leads to PAg-binding to the B30.2 domain of BTN3A1, and subsequent binding of the B30.2-PAg complex to the intracellular domain of BTN2A1. The resulting complex might include additional proteins, which finally bind all CDRs regions of γ and δ chain and trigger Vγ9Vδ2 T cell activation. Please note that so far neither direct binding of the TCR to BTN3A nor existence of an additional ligand recruited by the PAg-binding BTN3A has been demonstrated.

The Wilcox group in Birmingham, UK, demonstrated that the Vγ9 part of the TCR binds to BTN2A1 with a similar topology as BTNL3 to Vγ4 (24), which does not involve the TCRδ chain (11). Independently and with another screening system, the groups of Godfrey and Uldrich, at the University of Melbourne, also identified BTN2A1 as a key compound of PAg presentation. Both groups came to very similar conclusions on the interaction of BTN3A1, BTN2A1 and Vγ9Vδ2TCR (11, 12). However, the ligand(s) of the Vγ9Vδ2TCR in PAg-induced activation is still not known, it is speculated that it could be a particular conformation of BTN2A1 and BTN3A1. Our working hypothesis is that apart from BTN2A1 and BTN3A1, other molecules might be involved and that interaction of the TCR with these molecules implies also additional CDR3-binding ligand(s) which leads to different signals than exclusive binding of BTN(L) molecules to the Vγ part of the TCR (11) (**Figure 2**) 2). In summary, BTN and BTN-like molecules are essential for the development and TCR-mediated activation of many, if not all, γδ T cells.

## Other Function of Butyrophilins and Immune Therapy

BTN2A1 and BTN3A1 fulfill various functions. One function of BTN2A1 is its capacity to bind to DC-SIGN, which depends on the expressing cell type and the degree of its glycosylation state (31). An example of BTN3A1 function is its involvement in the induction of IFNβ production by cytoplasmic- or viral nucleic acids (32).

The immunomodulatory function of BTN(L) and using it as a target of immunotherapy is becoming of greater interest. For some time it has been known that BTN(L)-specific monoclonal antibodies can amplify activation of T cells and NK cells (5, 33), while BTN(L) overexpression, soluble BTN(L)-molecules or BTN(L)-Fc constructs often inhibit T-cell activation [e.g (2, 5)]. However, the involved counter receptors have not been identified and physiological relevance of this suppression is not clear. One of the better-understood examples about the physiological role of BTN(L)s comes from the analysis of BTN2a2-deficient mice. These show an increased αβT cell response, and shown by cell transfer experiments, this results from missing Btn2a2 expression of antigen-presenting cells (34). More recently, involvement was also reported in the regulation of ILC2-T cell crosstalk (35) and bone resorption (36) and reduced levels of soluble Btn2a2 in arthritis of in mouse or BTN2A2 in human arthritis (36).

A study which attracted much attention was the analysis of the suppression of tumor-specific T cells by BTN3A1 (37), which was postulated to be a consequence of BTN3A1-binding to glycosylated CD45 and concomitant disruption of the immunological synapse and TCR-mediated signaling. This immune suppression could be abolished by BTN3A specific monoclonal antibodies *in vitro* and in mouse models. Immunodeficient NGS mice were inoculated with a human ovarian cancer cell line and treated with combinations of human tumor-target specific TCR transductants. γδ T cells cells with and without BTN3 antibodies showed αβ and γδ T cell specific effects, by combining inhibition of BTN3-mediated suppression of the T cell response and agonistic action of agonist on the γδ T cells. The other model used was BTN3A1-transgenic mice inoculated with an immune suppression inducing ovarian tumor cell line (ID8-*Defb29*-*Vegf*-*a)*, where the therapeutic effect of the BTN3 specific mAb was even superior to PD1 specific mAb. Interestingly, even in absence of Vγ9Vδ2 T cells, administration of zoledronate had also a beneficial effect (37) which indicates that (partial) reversal of BTN3A1 immunosuppression might also involve PAgs. Another aspect is the interpretation of an ongoing clinical trial with the agonistic BTN3A specific monoclonal antibody ICT01 (ClinicalTrials.gov-Identifier: NCT04243499) where a positive clinical outcome might not only result from activation of Vγ9Vδ2 T cells (38) but also by reconstituting a BTN3-suppressed αβ T cell.

Very recently a mechanism of immune evasion by expression of BTNL2 on cancer cells was described, where BTNL2 promotes IL-17 production by a local γδ T cell population which enhances tumor resistance by recruitment of myeloid suppressor cells T cells (39). Blockade of BTNL2 by a monoclonal antibody had a significant therapeutic effect for several mouse tumors and acts synergistically with PD1 blockade. Furthermore, BTNL2 is expressed in multiple human solid cancers and its expression level correlates negatively with patients´ survival. Thus, BTNL2 may be a target for therapeutic intervention similar as BTN3 although the mechanism of immune evasion is a different one.

To conclude, BTN(L) molecules fulfill immunological and non-immunological functions of which some affect γδ T cells and bear therapeutic potential by targeting them with monoclonal antibodies similar to those successfully applied for more conventional B7 family members (40).

## Author Contributions

TH wrote the first version of the manuscript which was finalized together with MK. All authors contributed to the article and approved the submitted version.

## Funding

This study is funded by the DFG He 2346-8/2 which is part of the FOR 2799. This publication was supported by the Open Access Publication Fund of the University of Wuerzburg.

## Conflict of Interest

The authors declare that the research was conducted in the absence of any commercial or financial relationships that could be construed as a potential conflict of interest.

## Publisher’s Note

All claims expressed in this article are solely those of the authors and do not necessarily represent those of their affiliated organizations, or those of the publisher, the editors and the reviewers. Any product that may be evaluated in this article, or claim that may be made by its manufacturer, is not guaranteed or endorsed by the publisher.
